# Hiccough

**Published:** 1871-02

**Authors:** 


### HICCOUGH

Is a nervous contraction of certain muscles connected in their motions with the stomach. It is a fatal sign in some forms of disease, while cases are on record where the hiccough was so persistent as to induce convulsions and death. The old cure was "nine swallows of water," which, by distending the stomach, gave relief in many cases. But it is always desirable that the memory be stored with a variety of remedies for an affection which is so common, and at times inconvenient, to say the least of it. A lump or two of sugar, dissolved in the mouth and swallowed, has sometimes relieved the most distressing cases, and comes within the meaning of "The Food Cure," in "Health by Good Living."

